# Supplementary material for: Promoting the modal shift of freight from road to rail in China: An evolutionary game and simulation study
Source: PLoS One. 2025 May 23;20(5):e0320880. doi: 10.1371/journal.pone.0320880 (PMC12101862; doi:10.1371/journal.pone.0320880)
Supplement: S1 File — (DOCX) [file pone.0320880.s001.docx]

**Supporting Information**

All relevant data are within the manuscript.

After this article is published, it can be cited provided the source of the data is indicated.
