# Supplementary material for: Promoting the modal shift of freight from road to rail in China: An evolutionary game and simulation study
Source: PLoS One. 2025 May 23;20(5):e0320880. doi: 10.1371/journal.pone.0320880 (PMC12101862; doi:10.1371/journal.pone.0320880)
Supplement: Attachment — Submitted filename: PONE-D-24-54352_reviewer.pdf [file pone.0320880.s002.pdf]

PLOS ONE

Promoting the Modal Shift of Freight from Road to Rail in China An Evolutionary Game and Simulation Study

--Manuscript Draft--

**Manuscript Number:**	PONE-D-24-54352
**Article Type:**	Research Article
**Full Title:**	Promoting the Modal Shift of Freight from Road to Rail in China An Evolutionary Game and Simulation Study
**Short Title:**	Promoting the Modal Shift of Freight from Road to Rail in China An Evolutionary Game and Simulation Study
**Corresponding Author:**	Shuai Liu<br>China Academy of Railway Sciences<br>Beijing, CHINA
**Keywords:**	road to rail; railway freight transport; tripartite evolutionary game; simulation analysis
**Abstract:**	In the context of China's "Road to Rail" policy, this study constructs a tripartite evolutionary game model to mathematically investigate the strategic interactions among local governments, railway transport enterprises, and shippers. By employing mathematical modeling and simulation analysis, we examine the dynamic evolution of each stakeholder's strategy under varying conditions and verify the stability of the model. The findings indicate that government subsidies have a substantial effect on promoting shippers' adoption of rail transport, although this influence weakens over time. Furthermore, improvements in service quality by railway enterprises can significantly enhance the attractiveness of rail transport, with shippers demonstrating a capacity to swiftly adapt to changing market conditions. The research highlights that an initial phase of government subsidies, followed by their gradual withdrawal, can effectively facilitate the modal shift from road to rail by leveraging market mechanisms. This study provides theoretical insights and practical guidance for the formulation of effective transport policies, emphasizing the pivotal role of government incentives, the proactive engagement of railway enterprises, and the adaptive behavior of shippers in advancing sustainable freight transport.
**Order of Authors:**	Shuai Liu
	Guangzhi Jia
**Additional Information:**	
**Question**	**Response**
**Financial Disclosure**<br><br>Enter a financial disclosure statement that describes the sources of funding for the work included in this submission. Review the submission guidelines for detailed requirements. View published research articles from *PLOS ONE* for specific examples.<br><br>This statement is required for submission and **will appear in the published article** if the submission is accepted. Please make sure it is accurate.	The author(s) received no specific funding for this work.

Powered by Editorial Manager® and ProduXion Manager® from Aries Systems Corporation

**Funded studies**<br>Enter a statement with the following details:<br>• Initials of the authors who received each award<br>• Grant numbers awarded to each author<br>• The full name of each funder<br>• URL of each funder website<br>• Did the sponsors or funders play any role in the study design, data collection and analysis, decision to publish, or preparation of the manuscript?<br><br>Did you receive funding for this work?	
**Competing Interests**<br><br>Use the instructions below to enter a competing interest statement for this submission. On behalf of all authors, disclose any competing interests that could be perceived to bias this work—acknowledging all financial support and any other relevant financial or non-financial competing interests.<br><br>This statement is required for submission and **will appear in the published article** if the submission is accepted. Please make sure it is accurate and that any funding sources listed in your Funding Information later in the submission form are also declared in your Financial Disclosure statement.<br><br>View published research articles from *PLOS ONE* for specific examples.	The authors have declared that no competing interests exist.

Powered by Editorial Manager® and ProduXion Manager® from Aries Systems Corporation

**NO authors have competing interests**<br><br>Enter: *The authors have declared that no competing interests exist.*<br><br>**Authors with competing interests**<br><br>Enter competing interest details beginning with this statement:<br><br>*I have read the journal's policy and the authors of this manuscript have the following competing interests: [insert competing interests here]*<br><br>* typeset	
**Ethics Statement**<br><br>Enter an ethics statement for this submission. This statement is required if the study involved:<br><br>• Human participants<br>• Human specimens or tissue<br>• Vertebrate animals or cephalopods<br>• Vertebrate embryos or tissues<br>• Field research<br><br>Write "N/A" if the submission does not require an ethics statement.<br><br>General guidance is provided below. Consult the submission guidelines for detailed instructions. **Make sure that all information entered here is included in the Methods section of the manuscript.**	N/A

**Format for specific study types**<br><br>**Human Subject Research (involving human participants and/or tissue)**<br>• Give the name of the institutional review board or ethics committee that approved the study<br>• Include the approval number and/or a statement indicating approval of this research<br>• Indicate the form of consent obtained (written/oral) or the reason that consent was not obtained (e.g. the data were analyzed anonymously)<br><br>**Animal Research (involving vertebrate animals, embryos or tissues)**<br>• Provide the name of the Institutional Animal Care and Use Committee (IACUC) or other relevant ethics board that reviewed the study protocol, and indicate whether they approved this research or granted a formal waiver of ethical approval<br>• Include an approval number if one was obtained<br>• If the study involved *non-human primates*, add *additional details* about animal welfare and steps taken to ameliorate suffering<br>• If anesthesia, euthanasia, or any kind of animal sacrifice is part of the study, include briefly which substances and/or methods were applied<br><br>**Field Research**<br><br>Include the following details if this study involves the collection of plant, animal, or other materials from a natural setting:<br>• Field permit number<br>• Name of the institution or relevant body that granted permission	
**Data Availability**<br><br>Authors are required to make all data underlying the findings described fully available, without restriction, and from the time of publication. PLOS allows rare exceptions to address legal and ethical concerns. See the PLOS Data Policy and FAQ for detailed information.	Yes - all data are fully available without restriction

A Data Availability Statement describing where the data can be found is required at submission. Your answers to this question constitute the Data Availability Statement and **will be published in the article**, if accepted.<br><br>**Important:** Stating 'data available on request from the author' is not sufficient. If your data are only available upon request, select 'No' for the first question and explain your exceptional situation in the text box.<br><br>Do the authors confirm that all data underlying the findings described in their manuscript are fully available without restriction?	
**Describe where the data may be found in full sentences. If you are copying our sample text, replace any instances of XXX with the appropriate details.**<br><br>• If the data are **held or will be held in a public repository**, include URLs, accession numbers or DOIs. If this information will only be available after acceptance, indicate this by ticking the box below. For example: *All XXX files are available from the XXX database (accession number(s) XXX, XXX.).*<br>• If the data are all contained **within the manuscript and/or Supporting Information files**, enter the following: *All relevant data are within the manuscript and its Supporting Information files.*<br>• If neither of these applies but you are able to provide **details of access elsewhere**, with or without limitations, please do so. For example:<br><br>*Data cannot be shared publicly because of [XXX]. Data are available from the XXX Institutional Data Access / Ethics Committee (contact via XXX) for researchers who meet the criteria for access to confidential data.*<br><br>*The data underlying the results presented in the study are available from (include the name of the third party*	All relevant data are within the manuscript and its Supporting Information files.

and contact information or URL).<br>• This text is appropriate if the data are owned by a third party and authors do not have permission to share the data.<br><br>* typeset	
Additional data availability information:	

Powered by Editorial Manager® and ProduXion Manager® from Aries Systems Corporation

Manuscript Click here to access/download;Manuscript;paper.docx

Promoting the Modal Shift of Freight from Road to Rail in China An Evolutionary Game and Simulation Study

Shuai Liu[1], Guangzhi Jia[2]

(1. Postgraduate Department, China Academy of Railway Sciences, Beijing 100081, China;
2. Scientific & Technical Information Research Institute, China Academy of Railway Sciences Corporation Limited, Beijing 100081)

Correspondence: jiagzh@aliyun.com

Abstract: In the context of China's "Road to Rail" policy, this study constructs a tripartite evolutionary game model to mathematically investigate the strategic interactions among local governments, railway transport enterprises, and shippers. By employing mathematical modeling and simulation analysis, we examine the dynamic evolution of each stakeholder's strategy under varying conditions and verify the stability of the model. The findings indicate that government subsidies have a substantial effect on promoting shippers' adoption of rail transport, although this influence weakens over time. Furthermore, improvements in service quality by railway enterprises can significantly enhance the attractiveness of rail transport, with shippers demonstrating a capacity to swiftly adapt to changing market conditions. The research highlights that an initial phase of government subsidies, followed by their gradual withdrawal, can effectively facilitate the modal shift from road to rail by leveraging market mechanisms. This study provides theoretical insights and practical guidance for the formulation of effective transport policies, emphasizing the pivotal role of government incentives, the proactive engagement of railway enterprises, and the adaptive behavior of shippers in advancing sustainable freight transport.

Keywords: road to rail; railway freight transport; tripartite evolutionary game; simulation analysis

1 Introduction

Rail freight transport is widely recognized as a more environmentally sustainable mode of transportation due to its advantages of lower energy consumption, greater capacity, and reduced pollution[1, 2]. In contrast, road freight transport, while offering considerable flexibility and convenience, poses challenges related to high energy consumption, increased pollution, and road congestion.[3] Consequently, promoting a shift from road to rail for freight transport has become a crucial objective of transportation policies across many countries [4-6]. In line with this objective, the "Outline of the 14th Five-Year Plan for National Economic and Social Development and the Long-Range Objectives Through the Year 2035" of the People's Republic of China explicitly calls for accelerating the shift of bulk goods and medium to long-distance freight from road to rail.

Rail transport offers significant environmental and economic advantages, such as lower energy consumption, high capacity, and reduced pollution. However, it also faces limitations, such as insufficient flexibility and longer cargo turnover times, which hinder its ability to meet the needs of enterprises requiring fast, point-to-point transportation [7]. As a result, rail freight struggles to fully leverage its potential advantages, and its market share remains relatively low. To address this issue and enhance rail freight's market competitiveness, numerous scholars have conducted studies and provided valuable insights. Wang et al.[8] emphasized the need to integrate rail transport with logistics services, highlighting that a comprehensive logistics chain approach is essential. This involves not only addressing warehousing but also tackling vehicle marshalling, sorting, and delivery to achieve "door-to-door" logistics services. Zhang et al. [9] suggested that the railway sector could consider establishing its own road logistics companies, integrating drayage transport into the rail transport network, and introducing competitive mechanisms to improve quality and cost management in drayage services.

To further understand and effectively promote the shift in freight transport modes, extensive studies have been conducted both domestically and internationally. Lin [10] noted that rail freight volume is heavily influenced by policy direction, with significant fluctuations driven by policy changes. Feng [11] argued that accelerating the implementation of transport structure adjustment policies and promoting "road to rail" requires a combination of market dynamics and comprehensive governmental measures, including administrative, fiscal, and legal support. Rotaris L et al. [12] suggested that a modal shift from road transport to combined road-rail transport could have a substantial positive environmental impact. Wisetjindawat W et al. [13] observed that although road transport has a high market share in long-distance freight, strategies are needed to promote a shift

towards rail services. Shi et al. [14] found that when average train speeds exceed 50 km/h or loading times are shorter than 1.5 days, rail transport's market share exceeds 50%. Shi et al. [15] concluded that rail freight volume fluctuates in response to changes in the prices of other transport modes, while the rail rates themselves significantly influence rail freight demand—higher rates tend to decrease freight volume.

The "road to rail" model is also a form of multimodal transport, where integrating rail as a component of multimodal logistics is considered a key trend for future development. Numerous scholars have conducted studies in this area. For example, JT Borecka et al. [16] designed an optimized alternative service plan for a given set of line closures, including passenger rerouting, adjusted train schedules, bus bridging services, and additional train services. Ai et al. [17] developed a multi-objective optimization model that considers shippers' needs, the importance of timing in multimodal transport, and the carbon reduction advantages. Zhang et al. [18] proposed a multimodal transport route selection model centered on rail, incorporating time penalty costs and damage compensation costs, aiming for the lowest overall transportation cost while considering transport reliability and safety.

As the discussion of multimodal transport and its relevance to the "road to rail" strategy continues, it becomes important to consider how stakeholders adapt their strategies within evolving market conditions. Evolutionary game theory provides a valuable framework for understanding the strategic dynamics between key players. Evolutionary game theory considers the bounded rationality of multiple stakeholders involved in the game and focuses on how these stakeholders adapt and evolve their strategies in a dynamic environment [19]. Several studies have already been conducted in related fields. For example, Feng et al. [20] analyzed the strategy evolution in the China-Europe Railway Express market under subsidy withdrawal conditions. Yang et al. [21] emphasized the role of fiscal and tax subsidies as a regulatory tool by the government in balancing transport capacity and demand. Li et al. [22] suggested that a gradual withdrawal mechanism for government subsidies can promote coordination. Zhang et al. [23] analyzed the optimal strategies for competition and cooperation among nodal cities by comparing the demand, profit, and social welfare of platform companies under three scenarios: perfect competition, proactive platform cooperation, and government-led cooperation, with numerical analysis. Chen et al. [24] developed a game-theoretical model to analyze competition between maritime and rail transport. Tamannaei M et al. [25] constructed a game-theoretical model to study the competition between road transport and multimodal road-rail systems under government intervention.

Building upon the theoretical insights gained from evolutionary game theory, the innovation of this study lies in using simulation analysis to explore the evolution of multiple stakeholders' strategies and their impact on system stability. This approach helps address the limitations in previous research that lacked systematic analysis of multi-party interactions, providing a more comprehensive theoretical foundation and practical guidance for the effective implementation of the "road to rail" policy. By selecting local governments, railway transport enterprises, and shippers as the game participants, a tripartite evolutionary game model is constructed. Through simulation analysis of different strategy choices, the dynamic evolution of each party's strategy is examined, the validity of the model is verified, and the effectiveness of different strategies in promoting the shift from road to rail transport is evaluated. This study ultimately offers insights for the development of railway transport enterprises in China and supports the transition towards the "road to rail" model.

2 An Evolutionary Game Theoretical Model

2.1 Problem Description

The tripartite evolutionary game method effectively simulates the interactions among the government, railway transport enterprises, and shippers, capturing the strategy evolution of each party under bounded rationality and identifying the long-term stable state of the system through dynamic analysis. This method allows for the evaluation of different policies (such as government subsidies and service improvements by railway transport enterprises), aiding in the formulation of more practical policy combinations and promoting the shift in the transport structure from road to rail. The logical relationships among the tripartite evolutionary game participants constructed in this paper are illustrated in Figure 1.

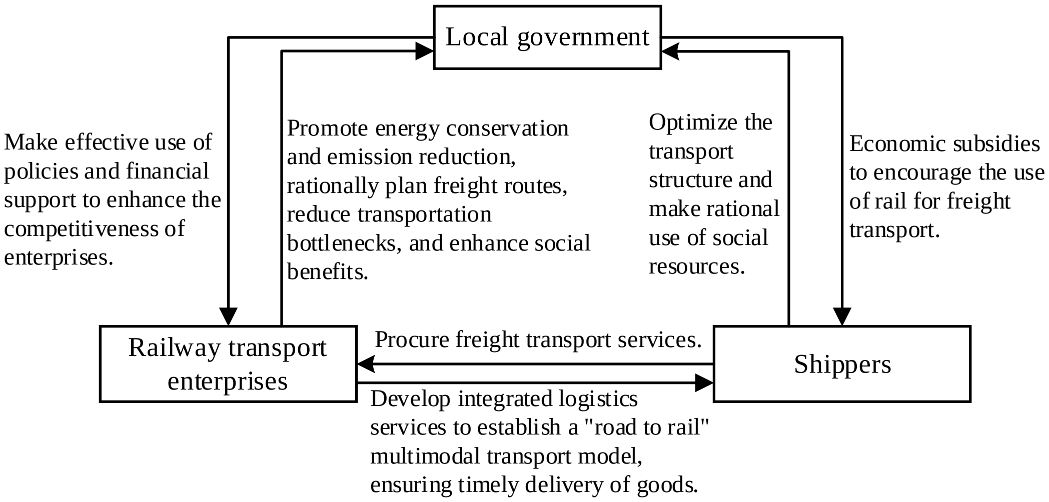

Figure 1. Logical Relationships Among Tripartite Evolutionary Game Participants

2.2. Model Assumptions and Notations

To facilitate the analysis of the constructed tripartite evolutionary game model, including the evolutionary strategies of each game participant, the interrelationships among the participants, and the stability analysis of strategy equilibrium points, the following assumptions are made:

Assumption 1: Local government, railway transport enterprises and shippers are considered the participants in the tripartite evolutionary game, each acting as a bounded rational agent aiming to maximize their own interests. The participants face information asymmetry, and their strategies evolve over time towards an optimal strategy.

Assumption 2: The strategy set of the local government is S_τ={S_{τ_1},S_{τ_2}}, where S_{τ_1} represents the strategy of providing subsidies to shippers, and S_{τ_2} represents the strategy of not providing subsidies. The strategy set of the railway transport enterprises is S_ω={S_{ω_1},S_{ω_2}}, where S_{ω_1} represents the strategy of actively cooperating, and S_{ω_2} represents the strategy of maintaining the status quo. The strategy set of the shippers is S_φ={S_{φ_1},S_{φ_2}}, where S_{φ_1} represents actively choosing rail transport as the mode of freight transport, and S_{φ_2} represents actively choosing road transport as the mode of freight transport.

Assumption 3: The probability that the local government chooses the strategy S_{τ_1} of providing subsidies to shippers is x, and the probability that it chooses the strategy S_{τ_2} of not providing subsidies is 1-x, where $x \in[0,1]$. The probability that railway transport enterprises choose the strategy S_{ω_1} of actively cooperating is y, and the probability that they choose the strategy S_{ω_2} of maintaining the status quo is 1-y, where $y \in[0,1]$. The probability that shippers actively choose rail as the mode of freight transport S_{φ_1} is z, while the probability of choosing road transport S_{φ_2} is 1-z, where $z \in[0,1]$.

Assumption 4:From the perspective of the local government, the total economic, environmental, and social benefits derived from transporting one unit of freight by road are denoted as G_{rh}. The total economic, environmental, and social benefits derived from transporting one unit of freight by rail are denoted as G_{rr}. The environmental and economic benefits resulting from shifting freight transport from road to rail are represented by G_t. The investment made by the local government in building new road-rail intermodal facilities to promote the shift is denoted as G_{ei}. The subsidy provided by the local government to shippers choosing rail as their mode of transport is represented by G_{es}. The cost of addressing additional energy consumption and environmental issues caused by road transport compared to rail transport is denoted as G_{ee}.

The local government has a certain probability δ, where $\delta \in [0,1]$, of supervising the shippers receiving subsidies. The cost of supervision is denoted as G_{cs}. It is assumed that any non-compliant behavior by shippers in receiving subsidies will certainly be detected. If shippers apply for subsidies but fail to carry out the related freight transport as required, the subsidy G_{es} will be recovered, and a penalty G_f will be imposed.

Assumption 5: From the perspective of railway transport enterprises, the operating cost is denoted as R_{ec}, and the profit is denoted as R_{rp}. When freight transport shifts from road to rail, the additional cost incurred is represented by R'_{ec}, and the additional profit is represented by R'_{rp}.

If the railway transport enterprises fail to complete the construction of new road-rail intermodal facilities, there is a certain probability ε, where $\varepsilon \in [0,1]$, that due to issues such as capacity constraints, the freight will not be delivered on time, resulting in compensation R_f paid to shippers.

Assumption 6: From the perspective of shippers, all goods can be transported by either rail or road, with the total quantity of goods being Q. Currently, the proportion of goods transported by rail is Q_1, and the proportion transported by road is Q_2, with the relationship given by:

$$Q = Q_1 + Q_2$$

After subsidies, if the total quantity of goods transported by rail is Q'_1 and by road is Q'_2, the relationship is:

$$Q = Q'_1 + Q'_2$$

For shippers, the total cost of transporting one unit of freight by rail is S_{pr}, while the cost of transporting one unit by road is S_{ph}. Compared to road transport, rail transport, when capacity is sufficient, can handle larger quantities of the same type of goods per shipment, resulting in savings in time and economic profit, represented by S_e.

2.3. Modeling Framework

Based on the above assumptions, the Payoff Matrix for the local government, railway transport enterprises, and shippers is constructed as shown in Table 1

Table 1. Payoff Matrix

Tripartite Evolutionary Game Strategies	$\boldsymbol{S_{\tau_1}}$:Local government provides subsidies to shippers		$\boldsymbol{S_{\tau_2}}$:Local government does not provide subsidies to shippers	
	S_{ω_1}:Railway transport enterprises actively cooperate	S_{ω_2}:Railway transport enterprises maintain the status quo	S_{ω_1}:Railway transport enterprises actively cooperate	S_{ω_2}:Railway transport enterprises maintain the status quo
$\boldsymbol{S_{\varphi_1}}$:Shippers actively choose rail transport	$G_{rr}Q_1' + G_{rh}Q_2' + G_t - G_{ei} - G_{es} - G_{cs} + \delta(G_{es} + G_f)$	$G_{rr}Q_1' + G_{rh}Q_2' + G_t - G_{es} - G_{cs} + \delta(G_{es} + G_f)$	$G_{rr}Q_1' + G_{rh}Q_2' + G_t - G_{ei}$	$G_{rr}Q_1' + G_{rh}Q_2' + G_t$
	$R_{rp} - R_{ec} + R_{rp}' - R_{ec}' + G_{ei}$	$R_{rp} - R_{ec} + R_{rp}' - R_{ec}' - \varepsilon R_f$	$R_{rp} - R_{ec} + R_{rp}' - R_{ec}' + G_{ei}$	$R_{rp} - R_{ec} - \varepsilon R_f$
	$G_{es} - Q_1'S_{pr} - Q_2'S_{ph} - \delta\left(G_{es} + G_f\right) + S_e$	$G_{es} - Q_1'S_{pr} - Q_2'S_{ph} - \delta\left(G_{es} + G_f\right) + \varepsilon R_f$	$-Q_1'S_{pr} - Q_2'S_{ph}$	$-Q_1'S_{pr} - Q_2'S_{ph} + \varepsilon R_f$
$\boldsymbol{S_{\varphi_2}}$:Shippers actively choose road transport	$G_{rr}Q_1 + G_{rh}Q_2 - G_{ei} - G_{ee} - G_{cs}$	$G_{rr}Q_1 + G_{rh}Q_2 - G_{ee} - G_{cs}$	$G_{rr}Q_1 + G_{rh}Q_2 - G_{ei} - G_{ee}$	$G_{rr}Q_1 + G_{rh}Q_2 - G_{ee}$
	$R_{rp} - R_{ec} + G_{ei}$	$R_{rp} - R_{ec} - \varepsilon R_f$	$R_{rp} - R_{ec} + G_{ei}$	$R_{rp} - R_{ec} - \varepsilon R_f$
	$-Q_1S_{pr} - Q_2S_{ph}$	$-Q_1S_{pr} - Q_2S_{ph} + \varepsilon R_f$	$-Q_1S_{pr} - Q_2S_{ph}$	$-Q_1S_{pr} - Q_2S_{ph} + \varepsilon R_f$

3 Model Solution and Analysis

3.1 Stability Analysis of Local Government's Strategies

By analyzing the stability of local government strategies, the evolutionary process of government behavior under different subsidy policies can be observed. Whether subsidies are provided or not, the government's strategic choices significantly influence the behavior of shippers and railway transport enterprises during the game process. This not only provides a basis for the formulation of government policies but also reveals its key role in promoting the "road to rail" transition. The expected payoff and the calculation of the average expected payoff for the local government are as follows.

The expected payoff E_{τ_1} for the local government when choosing the strategy S_{τ_1} of providing subsidies to shippers is:

$$E_{\tau_1} = yz[G_{rr}Q_1' + G_{rh}Q_2' + G_t - G_{ei} - G_{es} - G_{cs} + \delta(G_{es} + G_f) \quad]+ \quad y(1-z)(G_{rr}Q_1 + G_{rh}Q_2 - G_{ei} - G_{ee} - G_{cs} \ )+ \ (1-y)z[G_{rr}Q_1' + G_{rh}Q_2' + G_t - G_{es} - G_{cs} + \delta(G_{es} + G_f)] + (1-y)(1-z)(G_{rr}Q_1 + G_{rh}Q_2 - G_{ee} - G_{cs})$$

The expected payoff E_{τ_2} for the local government when choosing the strategy S_{τ_2} of not providing subsidies to shippers is:

$$E_{\tau_2} = yz(G_{rr}Q_1' + G_{rh}Q_2' + G_t - G_{ei}) + y(1-z)(G_{rr}Q_1 + G_{rh}Q_2 - G_{ei} - G_{ee}) \quad + \quad (1-y)z(G_{rr}Q_1' + G_{rh}Q_2') + (1-y)(1-z)(G_{rr}Q_1 + G_{rh}Q_2 - G_{ee})$$

The average payoff for the local government, denoted as $\bar{E}_\tau$, is:

$$\bar{E}_\tau = xE_{\tau_1} + (1-x)E_{\tau_2}$$

The replicator dynamic equation for the local government is:

$$\begin{aligned} F(x) = \frac{dx}{dt} &= x\left(E_{\tau_1} - \bar{E}_\tau\right) = x\left[E_{\tau_1} - xE_{\tau_1} + (1-x)E_{\tau_2}\right] = x(1-x)\left(E_{\tau_1} - E_{\tau_2}\right) \\ &= x(1-x)\{yz[G_{rr}Q_1' + G_{rh}Q_2' + G_t - G_{ei} - G_{es} - G_{cs} + \delta(G_{es} + G_f)] + y(1 \\ &\quad - z)(G_{rr}Q_1 + G_{rh}Q_2 - G_{ei} - G_{ee} - G_{cs}) + (1-y)z[G_{rr}Q_1' + G_{rh}Q_2' + G_t \\ &\quad - G_{es} - G_{cs} + \delta(G_{es} + G_f)] + (1-y)(1-z)(G_{rr}Q_1 + G_{rh}Q_2 - G_{ee} - G_{cs}) \\ &\quad - yz(G_{rr}Q_1' + G_{rh}Q_2' + G_t - G_{ei}) - y(1-z)(G_{rr}Q_1 + G_{rh}Q_2 - G_{ei} - G_{ee}) \\ &\quad - (1-y)z(G_{rr}Q_1' + G_{rh}Q_2') - (1-y)(1-z)(G_{rr}Q_1 + G_{rh}Q_2 - G_{ee})\} \end{aligned}$$

The first partial derivative of $F(x)$ with respect to x is:

$$\begin{aligned} \frac{dF(x)}{dx} &= (1-2x)\{yz[G_{rr}Q_1' + G_{rh}Q_2' + G_t - G_{ei} - G_{es} - G_{cs} + \delta(G_{es} + G_f)] + y(1 \\ &\quad - z)(G_{rr}Q_1 + G_{rh}Q_2 - G_{ei} - G_{ee} - G_{cs}) + (1-y)z[G_{rr}Q_1' + G_{rh}Q_2' + G_t \\ &\quad - G_{es} - G_{cs} + \delta(G_{es} + G_f)] + (1-y)(1-z)(G_{rr}Q_1 + G_{rh}Q_2 - G_{ee} - G_{cs}) \\ &\quad - yz(G_{rr}Q_1' + G_{rh}Q_2' + G_t - G_{ei}) - y(1-z)(G_{rr}Q_1 + G_{rh}Q_2 - G_{ei} - G_{ee}) \\ &\quad - (1-y)z(G_{rr}Q_1' + G_{rh}Q_2') - (1-y)(1-z)(G_{rr}Q_1 + G_{rh}Q_2 - G_{ee})\} \\ &= (1-2x)\{-G_{cs} + z[(\delta - 1)G_{es} + \delta G_f + (1-y)G_t]\} \end{aligned}$$

Let $-G_{cs} + z[(\delta - 1)G_{es} + \delta G_f + (1-y)G_t] = 0$,and solving for z^*, we get:

$$z^* = \frac{G_{cs}}{-yG_t - G_{es} + \delta G_{es} + \delta G_f + G_t}$$

When $z = z^*$, $F(x) \equiv 0$, and all values of x are in a stable state. When$0 \le z \le z^* \le 1$, $\frac{dF(x)}{dx}|_{x=0} > 0$ and $\frac{dF(x)}{dx}|_{x=1} < 0$, indicating that the equilibrium point is $x = 1$. Similarly, when $0 \le z^* \le z \le 1$, $\frac{dF(x)}{dx}|_{x=0} < 0$ and $\frac{dF(x)}{dx}|_{x=1} > 0$, indicating that the equilibrium point is $x = 0$. The phase diagram of the evolutionary behavior of government strategies is shown in Figure 2.

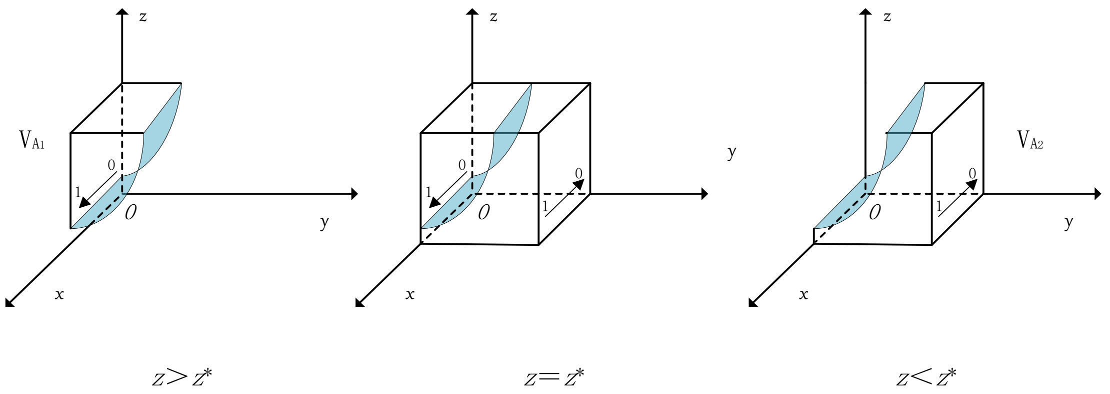

Figure 2. Phase Diagram of Local Government's Strategy Evolution

3.2 Stability Analysis of Railway Transport Enterprises' Strategies

The stability analysis of railway transport enterprises' strategies reveals their behavioral evolution under different market environments and policy conditions. When market demand increases or when government provides policy incentives, railway transport enterprises gradually tend to actively cooperate by optimizing service quality to enhance competitiveness. Although this proactive market response may take a longer time to yield significant effects, once enterprises increase investment and improve service capabilities, shippers' preference will also shift towards rail transport. The expected payoff and the calculation of the average expected payoff for railway transport enterprises are as follows.

The expected payoff E_{ω_1} for railway transport enterprises when choosing the strategy S_{ω_1} of actively cooperating is:

$$E_{\omega_1} = xz(R_{rp} - R_{ec} + R'_{rp} - R'_{ec} + G_{ei}) + x(1-z)(R_{rp} - R_{ec} + G_{ei}) + (1-x)z(R_{rp} - R_{ec} + R'_{rp} - R'_{ec} + G_{ei}) + (1-x)(1-z)(R_{rp} - R_{ec} + G_{ei})$$

The expected payoff E_{ω_2} for railway transport enterprises when choosing the strategy S_{ω_2} of maintaining the status quo is:

$$E_{\omega_2} = xz(R_{rp} - R_{ec} + R'_{rp} - R'_{ec} - \varepsilon R_f) + x(1-z)(R_{rp} - R_{ec} - \varepsilon R_f) + (1-x)z(R_{rp} - R_{ec} - \varepsilon R_f) + (1-x)(1-z)(R_{rp} - R_{ec} - \varepsilon R_f)$$

The average payoff for railway transport enterprises, denoted as $\bar{E}_\omega$, is:

$$\bar{E}_\omega = yE_{\omega_1} + (1-y)E_{\omega_2}$$

The replicator dynamic equation for railway transport enterprises is:

$$\begin{aligned} F(y) = \frac{dy}{dt} &= y(E_{\omega_1} - \bar{E}_\omega) = y[E_{\omega_1} - yE_{\omega_1} - (1-y)E_{\omega_2}] = y(1-y)(E_{\omega_1} - E_{\omega_2}) \\ &= y(1-y)\{xz(R_{rp} - R_{ec} + R'_{rp} - R'_{ec} + G_{ei}) + x(1-z)(R_{rp} - R_{ec} + G_{ei}) \\ &+ (1-x)z(R_{rp} - R_{ec} + R'_{rp} - R'_{ec} + G_{ei}) + (1-x)(1-z)(R_{rp} - R_{ec} + G_{ei}) \\ &- xz(R_{rp} - R_{ec} + R'_{rp} - R'_{ec} - \varepsilon R_f) - x(1-z)(R_{rp} - R_{ec} - \varepsilon R_f) \\ &- (1-x)z(R_{rp} - R_{ec} - \varepsilon R_f) - (1-x)(1-z)(R_{rp} - R_{ec} - \varepsilon R_f)\} \end{aligned}$$

The first partial derivative of $F(y)$ with respect to y is:

$$\begin{aligned} \frac{dF(y)}{dy} &= (1-2y)\{xz(R_{rp} - R_{ec} + R'_{rp} - R'_{ec} + G_{ei}) + x(1-z)(R_{rp} - R_{ec} + G_{ei}) \\ &+ (1-x)z(R_{rp} - R_{ec} + R'_{rp} - R'_{ec} + G_{ei}) + (1-x)(1-z)(R_{rp} - R_{ec} + G_{ei}) \\ &- xz(R_{rp} - R_{ec} + R'_{rp} - R'_{ec} - \varepsilon R_f) - x(1-z)(R_{rp} - R_{ec} - \varepsilon R_f) \\ &- (1-x)z(R_{rp} - R_{ec} - \varepsilon R_f) - (1-x)(1-z)(R_{rp} - R_{ec} - \varepsilon R_f)\} \\ &= (1-2y)[G_{ei} + \varepsilon R_f + (1-x)z(R'_{rp} - R'_{ec})] \end{aligned}$$

Let $G_{ei} + \varepsilon R_f + (1-x)z(R'_{rp} - R'_{ec}) = 0$,and solving for x^*, we get:

$$x^* = 1 + \frac{G_{ei} + \varepsilon R_f}{z(R'_{rp} - R'_{ec})}$$

When $x = x^*$, $F(y) \equiv 0$, and all values of y are in a stable state. When$0 \leq x \leq x^* \leq 1$, $\frac{dF(y)}{dy}|_{y=0} < 0$ and $\frac{dF(y)}{dy}|_{y=1} > 0$, indicating that the equilibrium point is $y = 0$. Similarly, when $0 \leq x^* \leq x \leq 1$, $\frac{dF(y)}{dy}|_{y=0} > 0$ and $\frac{dF(y)}{dy}|_{y=1} < 0$, indicating that the equilibrium point is $y = 1$. The phase diagram of the evolutionary behavior of railway transport enterprises strategies is shown in Figure 3.

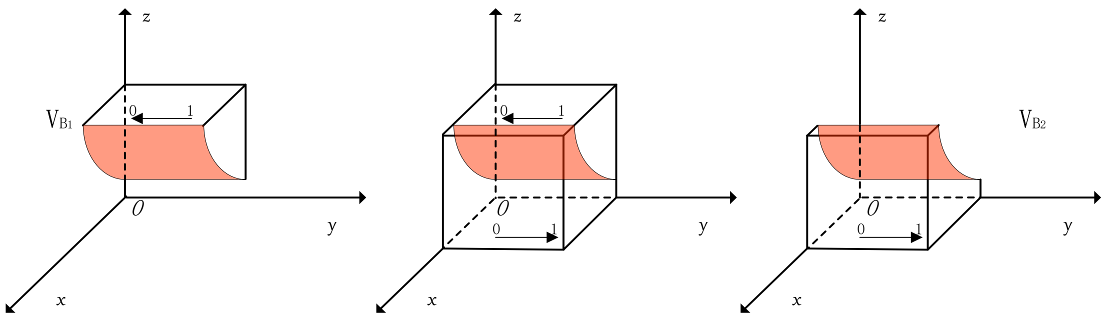

$x<x^*$ $x=x^*$ $x>x^*$

Figure 3 Phase Diagram of Railway Transport Enterprises' Behavior Evolution

3.3 Stability Analysis of Shippers' Strategies

As a key participant in the game, the stability analysis of shippers' strategies plays an important role in overall system stability. Shippers' strategy choices not only directly affect the proportion of rail and road transport but also have a feedback effect on the strategic adjustments of local government and railway enterprises. The expected payoff and the calculation of the average expected payoff for shippers are as follows.

The expected payoff E_{φ_1} for shippers when choosing the strategy S_{φ_1} of actively choosing rail transport is:

$$E_{\varphi_1} = xy[G_{es} - Q'_1 S_{pr} - Q'_2 S_{ph} - \delta(G_{es} + G_f) + S_e] + (1-x)y[G_{es} - Q'_1 S_{pr} - Q'_2 S_{ph} - \delta(G_{es} + G_f) + \varepsilon R_f] + x(1-y)(-Q'_1 S_{pr} - Q'_2 S_{ph}) + (1-x)(1-y)(-Q'_1 S_{pr} - Q'_2 S_{ph} + \varepsilon R_f)$$

The expected payoff E_{φ_2} for shippers when choosing the strategy S_{φ_2} of actively choosing road transport is:

$$E_{\varphi_2} = xy(-Q_1 S_{pr} - Q_2 S_{ph}) + (1-x)y(-Q_1 S_{pr} - Q_2 S_{ph} + \varepsilon R_f) + x(1-y)(-Q_1 S_{pr} - Q_2 S_{ph}) + (1-x)(1-y)(-Q_1 S_{pr} - Q_2 S_{ph} + \varepsilon R_f)$$

The average payoff for shippers, denoted as $\bar{E}_\varphi$, is:

$$\bar{E}_\varphi = zE_{\varphi_1} + (1-z)E_{\varphi_2}$$

The replicator dynamic equation for shippers is:

$$F(z)=\frac{dz}{dt}=z\left(E_{\varphi_1}-\bar{E}_{\varphi}\right)=z\left[E_{\varphi_1}-zE_{\varphi_1}-(1-z)E_{\varphi_2}\right]=z(1-z)\left(E_{\varphi_1}-E_{\varphi_2}\right)$$
$$=z(1-z)\{xy\left[G_{es}-Q_1'S_{pr}-Q_2'S_{ph}-\delta\left(G_{es}+G_f\right)+S_e\right]$$
$$+(1-x)y\left[G_{es}-Q_1'S_{pr}-Q_2'S_{ph}-\delta\left(G_{es}+G_f\right)+\varepsilon R_f\right]$$
$$+x(1-y)\left(-Q_1'S_{pr}-Q_2'S_{ph}\right)+(1-x)(1-y)\left(-Q_1'S_{pr}-Q_2'S_{ph}+\varepsilon R_f\right)$$
$$-xy\left(-Q_1S_{pr}-Q_2S_{ph}\right)-(1-x)y\left(-Q_1S_{pr}-Q_2S_{ph}+\varepsilon R_f\right)$$
$$-x(1-y)\left(-Q_1S_{pr}-Q_2S_{ph}\right)-(1-x)(1-y)(-Q_1S_{pr}-Q_2S_{ph}+\varepsilon R_f)\}$$

The first partial derivative of $F(z)$ with respect to z is:

$$\frac{dF(z)}{dt}=(1-2z)\{xy\left[G_{es}-Q_1'S_{pr}-Q_2'S_{ph}-\delta\left(G_{es}+G_f\right)+S_e\right]$$
$$+(1-x)y\left[G_{es}-Q_1'S_{pr}-Q_2'S_{ph}-\delta\left(G_{es}+G_f\right)+\varepsilon R_f\right]$$
$$+x(1-y)\left(-Q_1'S_{pr}-Q_2'S_{ph}\right)+(1-x)(1-y)\left(-Q_1'S_{pr}-Q_2'S_{ph}+\varepsilon R_f\right)$$
$$-xy\left(-Q_1S_{pr}-Q_2S_{ph}\right)-(1-x)y\left(-Q_1S_{pr}-Q_2S_{ph}+\varepsilon R_f\right)$$
$$-x(1-y)\left(-Q_1S_{pr}-Q_2S_{ph}\right)-(1-x)(1-y)\left(-Q_1S_{pr}-Q_2S_{ph}+\varepsilon R_f\right)\}$$
$$=(1-2z)[(1-\delta)yG_{es}-\delta yG_f+xyS_e+Q_2S_{ph}-Q_2'S_{ph}+Q_1S_{pr}-Q_1'S_{pr}]$$

Let $(1-\delta)yG_{es}-\delta yG_f+xyS_e+Q_2S_{ph}-Q_2'S_{ph}+Q_1S_{pr}-Q_1'S_{pr}=0$, and solving for y^*, we get:

$$y^*=\frac{Q_2S_{ph}-Q_2'S_{ph}+Q_1S_{pr}-Q_1'S_{pr}}{-G_{\mathrm{es}}+\delta G_{\mathrm{es}}+\delta G_f-xS_e}$$

When $y=y^*$, $F(z)\equiv 0$, and all values of z are in a stable state. When$0\le y\le y^*\le 1$, $\frac{dF(z)}{dz}|_{z=0}>0$ and $\frac{dF(z)}{dz}|_{z=1}<0$, indicating that the equilibrium point is $z=1$. Similarly, when $0\le y^*\le y\le 1$, $\frac{dF(z)}{dz}|_{z=0}<0$ and $\frac{dF(z)}{dz}|_{z=1}>0$, indicating that the equilibrium point is $z=0$. The phase diagram of the evolutionary behavior of shipper strategies is shown in Figure 4.

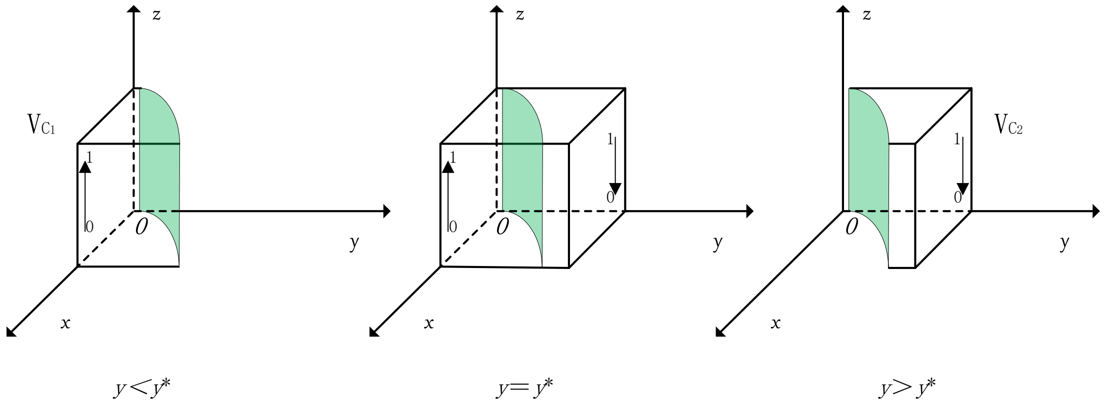

Figure 4 Phase Diagram of Shippers' Strategy Evolution

3.4 Stability Analysis of Equilibrium Points in the Tripartite Evolutionary Game System

The above three subsections respectively analyzed the strategy evolution processes of local government, railway transport enterprises, and shippers as individual participants. However, the tripartite evolutionary game system is the result of the combined evolution of strategies from all three parties. Therefore, by combining the dynamic equations of local government, railway transport enterprises, and shippers, we obtain:

$$\begin{cases} F(x) = x(1-x)\{-G_{cs} + z[(\delta-1)G_{es} + \delta G_f + (1-y)G_t]\} \\ F(y) = y(1-y)[G_{ei} + \varepsilon R_f + (1-x)z(R'_{rp} - R'_{ec})] \\ F(z) = z(1-z)[(1-\delta)yG_{es} - \delta yG_f + xyS_e + Q_2S_{ph} - Q'_2S_{ph} + Q_1S_{pr} - Q'_1S_{pr}] \end{cases}$$

The Jacobian matrix of the tripartite evolutionary game system is:

$$J = \begin{bmatrix} J_1 & J_2 & J_3 \\ J_4 & J_5 & J_6 \\ J_7 & J_8 & J_9 \end{bmatrix} = \begin{bmatrix} \frac{\partial F(x)}{\partial x} & \frac{\partial F(x)}{\partial y} & \frac{\partial F(x)}{\partial z} \\ \frac{\partial F(y)}{\partial x} & \frac{\partial F(y)}{\partial y} & \frac{\partial F(y)}{\partial z} \\ \frac{\partial F(z)}{\partial x} & \frac{\partial F(z)}{\partial y} & \frac{\partial F(z)}{\partial z} \end{bmatrix}$$

The pure strategy equilibrium points are $E_1(0,0,0)$, $E_2(0,0,1)$, $E_3(0,1,0)$, $E_4(1,0,0)$, $E_5(0,1,1)$, $E_6(1,0,1)$, $E_7(1,1,0)$, $E_8(1,1,1)$;The mixed strategy equilibrium points are $E_9(x^*,0,0)$,$E_{10}(0,y^*,0)$,$E_{11}(0,0,z^*)$. A mixed strategy equilibrium point represents a non-evolutionarily stable strategy.**Error! Reference source not found.**

According to Lyapunov first method (indirect method), a pure strategy equilibrium point is a stable strategy if all eigenvalues of the constructed Jacobian matrix have negative real parts. If any eigenvalue has a positive real part, the equilibrium point is either unstable or indeterminate. The eigenvalues of the Jacobian matrix for pure strategy equilibrium points are shown in Table 2.

Table 2 Stability Analysis of Evolutionary Stable Strategy

**Evolutionary Stable Strategy**	**Eigenvalues of the Jacobian Matrix**	**Stability**
$E_1(0,0,0)$	$\lambda_1 = -G_{cs}$<br>$\lambda_2 = G_{ei} + \varepsilon R_f$<br>$\lambda_3 = Q_2S_{ph} - Q'_2S_{ph} + Q_1S_{pr} - Q'_1S_{pr}$	(-, +, +)
$E_2(0,0,1)$	$\lambda_1 = -G_{cs} - G_{es} + \delta G_{es} + \delta G_f + G_t$<br>$\lambda_2 = G_{ei} - R_{ec} + \varepsilon R_f + R'_{rp}$<br>$\lambda_3 = -Q_2S_{ph} + Q'_2S_{ph} - Q_1S_{pr} + Q'_1S_{pr}$	(-, ×, -)
$E_3(0,1,0)$	$\lambda_1 = -G_{cs}$<br>$\lambda_2 = -G_{ei} - \varepsilon R_f$<br>$\lambda_3 = G_{es} - \delta G_{es} - \delta G_f + Q_2S_{ph} - Q'_2S_{ph} + Q_1S_{pr} - Q'_1S_{pr}$	(-, -, +)
$E_4(1,0,0)$	$\lambda_1 = G_{cs}$	(+, +, +)

Evolutionary Stable Strategy	Eigenvalues of the Jacobian Matrix	Stability
	$\lambda_2 = G_{ei} + \varepsilon R_f$<br>$\lambda_3 = Q_2 S_{ph} - Q_2' S_{ph} + Q_1 S_{pr} - Q_1' S_{pr}$	
$E_5(0,1,1)$	$\lambda_1 = -G_{cs} - G_{es} + \delta G_{es} + \delta G_f$<br>$\lambda_2 = -G_{ei} + R_{rc}' - R_{rp}' - \varepsilon R_f$<br>$\lambda_3 = -G_{es} + \delta G_{es} + \delta G_f - Q_2 S_{ph} + Q_2' S_{ph} - Q_1 S_{pr} + Q_1' S_{pr}$	(-, ×, -)
$E_6(1,0,1)$	$\lambda_1 = G_{cs} + G_{es} - \delta G_{es} - \delta G_f - G_t$<br>$\lambda_2 = G_{ei} + \varepsilon R_f$<br>$\lambda_3 = -Q_2 S_{ph} + Q_2' S_{ph} - Q_1 S_{pr} + Q_1' S_{pr}$	(×, +, -)
$E_7(1,1,0)$	$\lambda_1 = G_{cs}$<br>$\lambda_2 = -G_{ei} - \varepsilon R_f$<br>$\lambda_3 = (1-\delta) G_{es} - \delta G_f + S_e + Q_2 S_{ph} - Q_2' S_{ph} + Q_1 S_{pr} - Q_1' S_{pr}$	(+, -, +)
$E_8(1,1,1)$	$\lambda_1 = G_{cs} + G_{es} - \delta G_{es} - \delta G_f$<br>$\lambda_2 = -G_{ei} - \varepsilon R_f$<br>$\lambda_3 = -G_{es} + \delta G_{es} + \delta G_f - S_e - Q_2 S_{ph} + Q_2' S_{ph} - Q_1 S_{pr} + Q_1' S_{pr}$	(×, -, -)

Note: "+" indicates a positive eigenvalue, "-" indicates a negative eigenvalue, and "×" indicates that the sign of the eigenvalue depends on the parameter values assigned.

Scenario 1:

When $G_{ei} - R_{ec} + \varepsilon R_f + R_{rp}' < 0$, all eigenvalues of the Jacobian matrix constructed at $E_2(0,0,1)$ have negative real parts, indicating a stable equilibrium point. The corresponding evolutionary game strategy is S_1={ local government does not provide subsidies to shippers, railway transport enterprises maintain the status quo, shippers actively choose rail transport}.

Analysis: This represents an ideal equilibrium state where the local government does not rely on fiscal incentives to drive transport adjustments, railway transport enterprises achieve profit through maintaining their existing operations, and shippers make an active choice due to the cost advantages or environmental benefits of rail transport. However, the sustainability of this equilibrium depends on changes in market conditions and the dynamic adjustments of each party's

behavior. Without sufficient incentives and reforms, the enthusiasm of shippers may gradually diminish, thereby affecting the competitiveness of rail transport.

Scenario 2:

When $G_{es} - \delta G_{es} - \delta G_f + Q_2 S_{ph} - Q_2' S_{ph} + Q_1 S_{pr} - Q_1' S_{pr} < 0$, all eigenvalues of the Jacobian matrix constructed at $E_3(0,1,0)$ have negative real parts, indicating a stable equilibrium point. The corresponding evolutionary game strategy is S_2={ local government does not provide subsidies to shippers, railway transport enterprises actively cooperate, shippers actively choose road transport}.

Analysis: The active cooperation of railway transport enterprises indicates that they have taken proactive measures to enhance their competitiveness in the market, striving to attract shippers to choose rail transport. However, shippers, based on market conditions and transportation costs, still tend to prefer road transport, likely due to the flexibility, convenience, and better adaptability of road transport to their freight needs.

This equilibrium point indicates that, under current conditions, the strategic combination of all parties has reached a relatively stable state. Even though railway enterprises take proactive measures, the lack of subsidy from the local government and the shippers' preference for road transport result in the overall game system gravitating towards this stable state.

Scenario 3:

When $-G_{ei} + R_{rc}' - R_{rp}' - \varepsilon R_f < 0$, all eigenvalues of the Jacobian matrix constructed at $E_5(0,1,1)$ have negative real parts, indicating a stable equilibrium point. The corresponding evolutionary game strategy is S_3={ local government does not provide subsidies to shippers, railway transport enterprises actively cooperate, shippers actively choose rail transport}.

Analysis: The formation of this stable state can be understood as the market forces in the multi-party game reaching a new balance. The local government chooses not to provide subsidies, and railway transport enterprises cannot rely on direct economic support from the government. Instead, they must attract shippers by enhancing their own market competitiveness. This improvement in competitiveness may manifest in various aspects, such as reducing freight rates, increasing transport efficiency, improving customer service, and expanding the service network.

On the other hand, shippers, after weighing various factors under current market conditions, decide to choose rail transport. This decision may be influenced by the benefits brought about by the active cooperation of railway transport enterprises, such as lower transport costs, faster delivery, and greater cargo security. Additionally, in some scenarios, rail transport holds unique advantages over road transport, and the choice of rail by shippers could be driven by considerations of their long-term interests.

Despite the absence of direct government subsidies, railway transport enterprises are still able to attract shippers through proactive market behavior. This active cooperation not only helps the enterprises gain market share but also strengthens their position in the market. In such a market environment, shippers, based on their needs and cost considerations, choose rail transport as the primary mode, thereby bringing the entire system to a relatively stable equilibrium state. This equilibrium point reflects that, even without government intervention, the market can achieve an effective balance through the self-adjustment of all parties involved.

Scenario 4:

When $G_{cs} + G_{es} - \delta G_{es} - \delta G_f < 0$, all eigenvalues of the Jacobian matrix constructed at $E_8(1,1,1)$ have negative real parts, indicating a stable equilibrium point. The corresponding

evolutionary game strategy is S_4={ local government provides subsidies to shippers, railway transport enterprises actively cooperate, shippers actively choose rail transport}.

Analysis: The local government reduces the cost of choosing rail transport for shippers through subsidies, incentivizing shippers to opt more for rail transport. This policy intervention effectively shifts shippers' transport preferences, encouraging more freight to be moved by rail. Railway transport enterprises actively cooperate with government policies by adopting measures to enhance their competitiveness, such as improving service quality and efficiency, further attracting shippers to choose rail transport. Under the dual effect of subsidies and proactive measures by railway enterprises, shippers find that the overall cost of choosing rail transport decreases while they also receive higher quality service, thus becoming more inclined to select rail transport.

This equilibrium point represents a cooperative relationship between railway transport enterprises and shippers under the incentive of government subsidies, forming a stable transport choice model. A positive feedback mechanism emerges among the local government, railway transport enterprises, and shippers, which reinforces itself and helps maintain the stability of this strategy combination.

4 Simulation Analysis

To verify the stability of the tripartite evolutionary game system, parameter values were assigned based on existing literature and relevant data, and numerical simulations of the model were conducted using Python 3.8.

4.1 Parameter Assignment

Based on relevant literature and data from a specific segment of China's railways, the parameters in the simulation are assigned as follows: according to the "Regulations on Railway Freight Transport," the compensation for goods is set as $R_f = 2000$;$R'_{rp} = 64$,$R'_{ec} = 16$,$S_e = 100$; $S_{pr} = 0.07$,$S_{ph} = 0.13$**Error! Reference source not found.** ;$\delta = 45\%$**Error! Reference source not found.**; according to the "China National Railway for the year 2023," $\varepsilon = 4\%$;$G_{es} = 2000$,$G_f = 20000$,$G_{ei} = 100$,$G_t = 30$,$Q_1 = 0.1$,$Q'_1 = 0.3$,$Q_2 = 0.9$,$Q'_2 = 0.7$.The model is then analyzed under these conditions.

4.2 Initial Evolutionary Path

To verify the stability of the tripartite evolutionary game, numerical simulations of the model were conducted using Python 3.8, with the above parameter assignments. The simulation step size was set to 0.2, and the initial value was set to 0.1. The simulation results are shown in Figure 5.

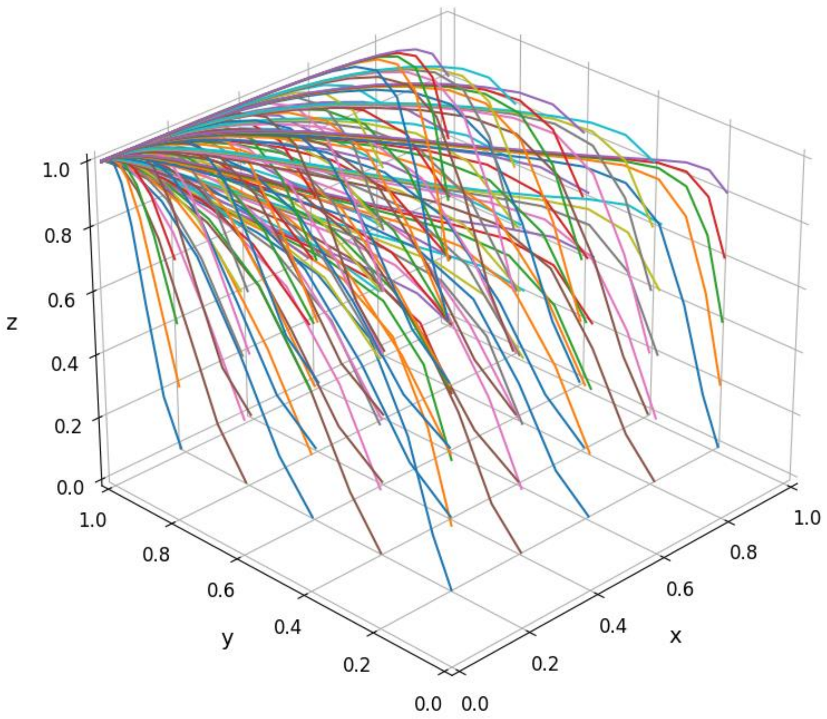

Figure 5. Initial Evolutionary Path Diagram

The simulation results indicate that, under the condition that all eigenvalues of the constructed Jacobian matrix have negative real parts, the equilibrium point for this set of data is (0,1,1). At this point, there is a stable strategy set for the system: {local government does not provide subsidies to shippers, railway transport enterprises actively cooperate, shippers actively choose rail transport}, which is consistent with the stability analysis results of the equilibrium point discussed above.

4.3 Initial Value Evolution Simulation Analysis

Under different initial values for x, y, and z, all subplots (a) to (e) in Figure 6 converge to the same stable state, with the corresponding evolutionary game strategy being S_3={ local government does not provide subsidies to shippers, railway transport enterprises actively cooperate, shippers actively choose rail transport} , consistent with the stability analysis of the equilibrium point discussed above.

In subplot (a), when the initial value is set as (0.2,0.2,0.2), government intervention has a certain influence in the early stages. However, this influence gradually diminishes over time, and shippers' strategy shifts towards rail transport, while the market share of railway transport enterprises grows steadily. Similarly, in subplots (b) to (f), although initial conditions vary and the evolutionary paths of the system differ slightly, the final equilibrium state remains fundamentally unchanged. Shippers still prefer a specific mode of transport, while the influence of government intervention gradually weakens and even disappears.

From Figure 6, it can be seen that the decision-making behavior of shippers evolves the fastest in this system, reaching a steady state first. Regardless of the initial conditions, shippers' decisions change rapidly in the early stages of evolution and stabilize within a short time. This indicates that shippers are strongly influenced by market conditions, costs, and transport efficiency when choosing a mode of transport, and they are able to make decisions quickly to adapt to a changing environment.

In contrast to the rapid decision-making of shippers, the behavior of railway transport enterprises evolves more slowly. Under different initial conditions, the market share of railway

transport enterprises gradually increases, reaching stability over a relatively longer period. This suggests that the measures taken by railway transport enterprises in response to market competition take longer to have an impact. Although the market share of railway transport enterprises eventually stabilizes and becomes dominant, the early adjustment process is relatively slow, indicating that these enterprises need more time to adjust strategies, optimize services, or enhance competitiveness in the face of market competition.

Initially, the local government may guide the market through policy intervention, allowing the market to achieve a self-regulating stable state under certain policy and competitive conditions. In the long run, enterprises and shippers in the market will adjust according to their own interests and market conditions, leading the system towards stability. During this process, the role of the local government gradually diminishes, and the market mechanism becomes the primary regulating force.

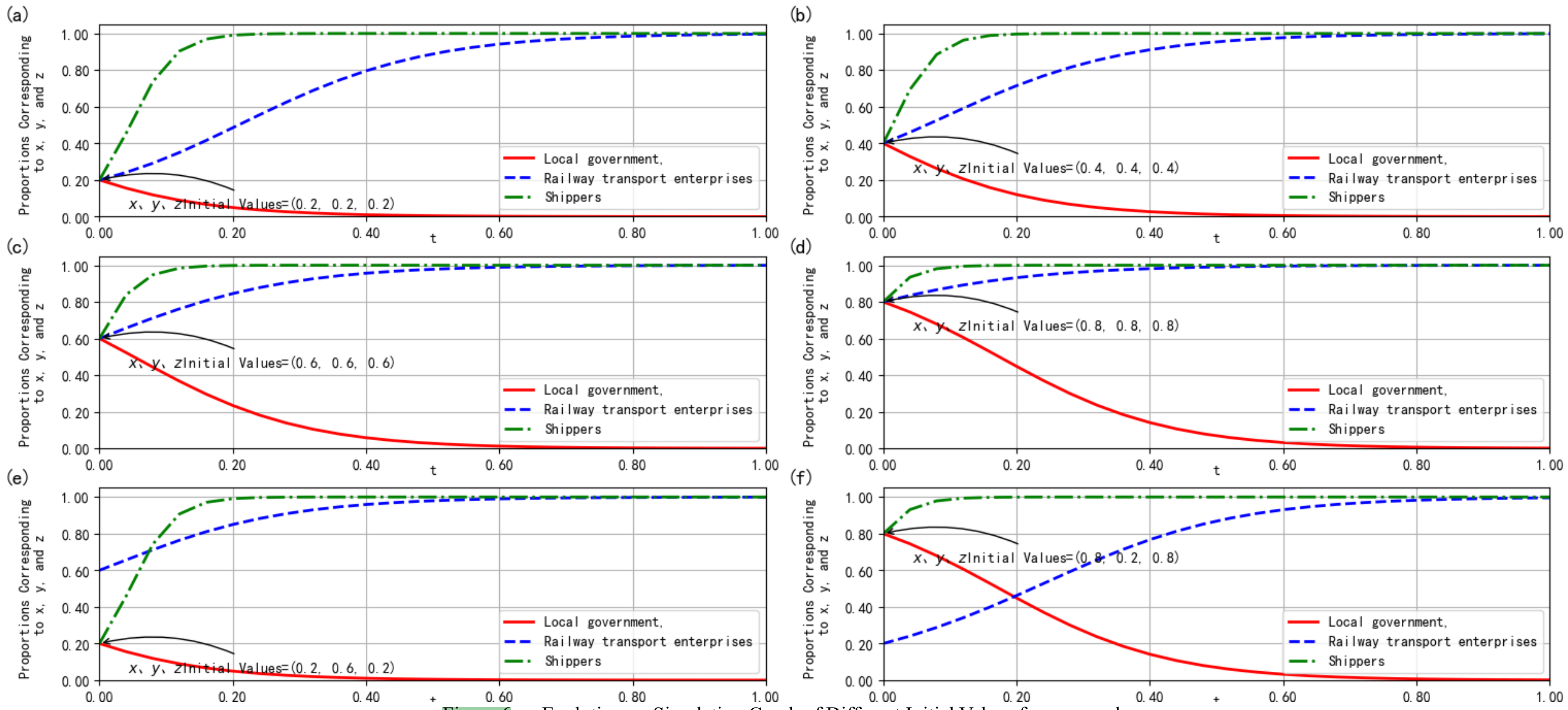

Figure 6 Evolutionary Simulation Graph of Different Initial Values for x, y, and z

4.4 Sensitivity Analysis of Simulation Parameters

To eliminate the impact of the initial values of selected parameters on the evolutionary game results of the system, the target parameters were selected for corresponding sensitivity analysis.

4.4.1 Sensitivity Simulation Analysis of the Road-Rail Freight Volume Ratio Parameters Q_1 and Q_2

To investigate the impact of road freight volume Q_1 and rail freight volume Q_2 on the evolutionary game results of the system, the parameters were set to different values for the road-rail freight volume ratio while keeping other parameters constant: $Q_1 = 10\%$、$Q_2 = 90\%$;$Q_1 = 20\%$、$Q_2 = 80\%$;$Q_1 = 30\%$、$Q_2 = 70\%$;$Q_1 = 40\%$、$Q_2 = 60\%$;$Q_1 = 50\%$、$Q_2 = 50\%$. The simulation results are shown in Figure 7.

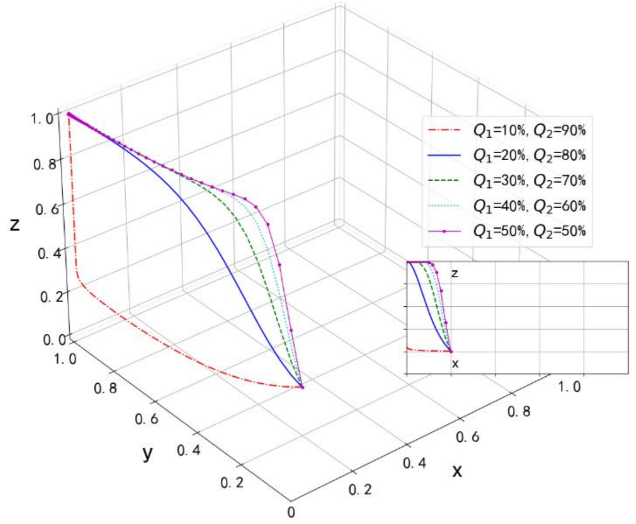

Figure 7. Sensitivity Analysis of Road-Rail Freight Volume Parameters Q_1 and Q_2

As shown in Figure 7, under different initial values for Q_1 and Q_2, the simulation ultimately converges to the same stable state, specifically the point (0,1,1), which is consistent with the stability analysis results of the equilibrium point discussed above.

Currently, the ratio of rail freight volume to road freight volume in China is approximately 1:9, and the convergence to a stable state in the evolutionary game is relatively slow. When the proportion of rail freight gradually increases, the system converges to a stable state more quickly. This indicates that in the current transport system, promoting the shift of freight transport from road to rail is necessary to enhance the overall efficiency of the system. Increasing the proportion of rail freight not only reduces overall transportation costs but also decreases carbon emissions and other environmental pollution, as well as reduces accidents and risks associated with road transport.

4.4.2 Sensitivity Simulation Analysis of Government Subsidy Parameter G_{es} for Shippers

To investigate the impact of government subsidies G_{es} for shippers on the evolutionary game results of the system, while keeping other parameters constant, the subsidy parameter G_{es} was assigned values of $G_{es} = 2000$、$G_{es} = 3000$、$G_{es} = 5000$、$G_{es} = 8000$、$G_{es} = 10000$. The simulation results are shown in Figure 8.

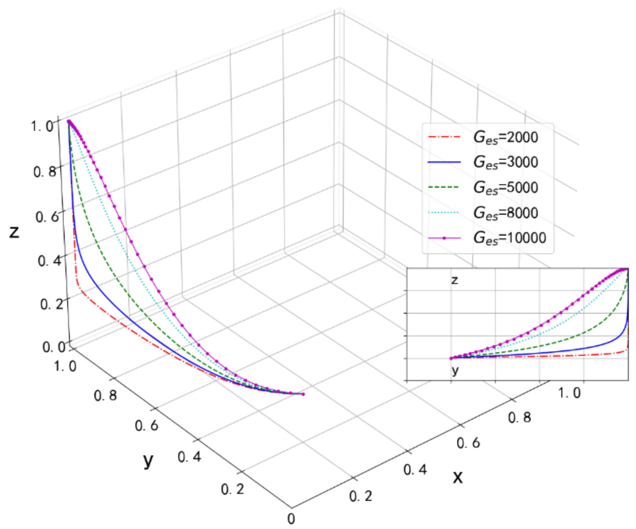

Figure 8 Sensitivity Analysis of Government Subsidy Parameter G_{es} for Shippers

As shown in Figure 8, under different initial values for G_{es}, the simulation ultimately converges to the same stable state, specifically the point (0,1,1), which is consistent with the stability analysis results of the equilibrium point discussed above.

The growth trend and turning points of the curve can reflect the stability and sensitivity of the system under different conditions. The curve corresponding to a lower subsidy value G_{es} tends to be lower in the range of changes for y and z, indicating that the system takes longer to reach an equilibrium state under lower stimuli. As the subsidy value G_{es} appropriately increases, the corresponding y and z values of the curve become higher, suggesting that the system reaches equilibrium more quickly. However, as G_{es} continues to increase, the speed at which the system reaches equilibrium begins to decrease, indicating that the marginal utility of higher subsidies diminishes compared to lower subsidy increments. In the early stages of market development, government subsidies are needed to influence the strategic behavior of shippers. However, as the system continues to evolve, the effectiveness of subsidies gradually decreases until eventually, the local government no longer provides subsidies to shippers, and the tripartite evolutionary game system reaches an equilibrium state.

5. Conclusions

In this study, a tripartite evolutionary game system involving local government, railway transport enterprises, and shippers was constructed. Based on real data collection, combined with theoretical analysis and simulation models, the stability of decision-making behaviors among the three parties was analyzed. The conclusions are as follows:

(1) **Shippers adjust decisions rapidly based on market changes**: The strategy evolution speed of shippers is significantly higher than that of local government and railway transport enterprises, reflecting shippers' high sensitivity and adaptability to market changes. This rapid adjustment is not only driven by shippers' direct interest in optimizing transportation efficiency and cost but also indicates their flexible response to policy changes. This quick response may have a dual impact on market competition: on one hand, it pushes railway transport enterprises to continuously optimize service quality and transportation prices to maintain an advantageous position in the game; on the other hand, it encourages local governments to adjust policy support in a timely manner, forming a dynamic evolution mechanism of mutual incentives among policies, enterprises, and the market, thus achieving the optimal goal of transport structure adjustment.

(2) **Low current rail freight volume, but a noticeable trend in absorbing road freight**: Although the current rail freight volume accounts for only about 10% of the total, with road transport taking a 90% share, the simulation results reveal an increasingly significant trend of freight shifting from road to rail under local government policy incentives. When the rail freight proportion rises to about 30%, the system reaches equilibrium more quickly. This shift not only effectively reduces overall transportation costs but also significantly decreases negative externalities such as carbon emissions and traffic accidents during transportation, especially bringing notable social benefits in environmental protection and public safety. Moreover, as rail freight increases, the economies of scale begin to emerge, further consolidating the competitive advantage of rail transport. This indicates that, through scientific policy guidance, the "road to rail" shift can reduce overall societal costs while optimizing the transport structure, creating a synergy of economic, environmental, and social benefits.

(3) **The importance of government subsidies and a phased withdrawal strategy**: In the initial evolution phase of the system, government subsidies for shippers play an essential role in encouraging the shift towards rail transport. Simulation analysis shows that increasing subsidy amounts (from 2000 to 5000 yuan) significantly accelerates system stability, making shippers more willing to choose rail transport. However, as the market matures and the collaboration and trust between railway transport enterprises and shippers strengthen, the market's self-regulation capacity also gradually increases. At this stage, the marginal benefit of high subsidies diminishes and may even lead to excessive dependence on subsidies, which is detrimental to the effective functioning of market mechanisms. Therefore, the government should provide appropriate and effective subsidies during the initial evolution of the system to establish trust and preference for rail transport in the market. As the system enters a stable phase, subsidies should be gradually reduced and eventually withdrawn to stimulate the market's self-regulatory capacity, ultimately achieving sustainable development in transport structure adjustment. This gradual withdrawal strategy helps to reduce fiscal burdens and prevents complacency among market players, motivating railway transport enterprises to win the market by improving service quality and efficiency.

6 Future Research Directions

(1) Extended Study of Participating Entities

This paper selects local governments, railway transport enterprises, and shippers as participants in the evolutionary game, but in reality, the participants may go beyond these three parties. For instance, logistics service providers, financial institutions, environmental organizations, and other stakeholders may also have a significant impact on the implementation of the "road to rail" strategy. Therefore, future research could consider incorporating more stakeholders into the game model to more comprehensively simulate real-world scenarios, thereby gaining a better understanding of interactions among stakeholders and their impact on transport structure adjustment.

(2) Optimization and Refinement of Subsidy Policies

For the subsidy policy for rail freight, further research is needed on how to gradually rationalize and optimize subsidies. While moderate financial support in the early stages of subsidy can effectively promote the shift of freight to rail, the challenge over the long term is how to gradually reduce subsidies, avoid reliance by market participants, and maintain the competitiveness of rail transport. Future research could involve constructing evolutionary game models of different subsidy strategies, evaluating the long-term effects of various subsidy policies, and proposing optimal paths for phasing out subsidies.

(3) Policy Support and Improvement of the Multimodal Transport Model

To effectively implement the "road to rail" strategy, the government needs to introduce more supportive policies to promote the improvement of the multimodal transport model. Future research could delve into the impact of government policies on the multimodal transport model, including policy measures related to infrastructure development, the establishment of information-sharing platforms, and tax incentives. Additionally, studying the collaboration mechanisms within the multimodal transport model and their role in enhancing overall transport efficiency could provide a theoretical basis for the government to develop scientific and rational policies.

(4) Proactiveness and Business Strategy Adjustment of Railway Transport Enterprises

Railway transport enterprises should take the initiative, actively exerting subjective dynamism by adjusting their business strategies and models to adapt to the ever-changing market demand, particularly in playing a larger role in promoting the "road to rail" strategy and multimodal transport. Future research could explore how railway transport enterprises can enhance competitiveness through service innovation, resource optimization, and flexible adaptation to market changes, thus better promoting the shift of freight from road to rail and advancing the development of multimodal transport. Case studies and empirical analyses could be used to summarize effective business strategies, providing guidance for enterprises to maintain competitiveness in the market-driven process.

References:

[1] GANDHI N, KANT R, THAKKAR J. A systematic scientometric review of sustainable rail freight transportation [J]. Environ Sci Pollut Res Int, 2022, 29(47): 70746-71.

[2] KLJAIĆ Z, PAVKOVIĆ D, CIPEK M, et al. An Overview of Current Challenges and Emerging Technologies to Facilitate Increased Energy Efficiency, Safety, and Sustainability of Railway Transport [J]. Future Internet, 2023, 15(11): 347.

[3] TURAN B, HEMMELMAYR V, LARSEN A, et al. Transition towards sustainable mobility: the role of transport optimization [J]. Central European Journal of Operations Research, 2024, 32(2): 435-56.

[4] BASK A, RAJAHONKA M. The role of environmental sustainability in the freight transport mode choice: A systematic literature review with focus on the EU [J]. International Journal of Physical Distribution & Logistics Management, 2017, 47(7): 560-602.

[5] KALLAS S. White Paper on Transport: Roadmap to a Single European Transport Area--towards a Competitive and Resource-efficient Transport System [M]. Office for Official Publications of the European Communities, 2011.

[6] KAACK L H, VAISHNAV P, MORGAN M G, et al. Decarbonizing intraregional freight systems with a focus on modal shift [J]. Environmental Research Letters, 2018, 13(8): 083001.

[7] FERRARI P. Some necessary conditions for the success of innovations in rail freight transport [J]. Transportation Research Part A: Policy and Practice, 2018, 118: 747-58.

[8] WANG D, LI X F, LI G. Research on Bottlenecks and Solutions for the Development of Railway Logistics in China [J]. China Railway, 2016, (6): 38-42.

[9] ZHANG R, SHEN Y F, CHU L C. The impact of railway freight timeliness on shippers' choice of transportation mode [J]. China Railway, 2015, (8): 15-8.

[10] LIN W T. A Study on the Development Strategies of Railway Freight Transportation with Implementation of the national Transportation Restructuring Policy of "Promoting Railway Instead of Highway Transportation" [J]. Railway Freight Transport, 2019, 8.
[11] FENG W B. A Study on the Influencing Factors and Strategies of the Optimal Adjustment of China's Transport Structure [J]. Railway Transport and Economy, 2019, 41(9): 18-23.
[12] ROTARIS L, TONELLI S, CAPOANI L. Combined transport: Cheaper and greener. A successful Italian case study [J]. Research in Transportation Business & Management, 2022, 43: 100792.
[13] WISETJINDAWAT W, OIWA H, FUJITA M. Rare Mode Choice in Freight Transport: Modal Shift from Road to Rail [J]. Journal of the Eastern Asia Society for Transportation Studies, 2015, 11: 774-87.
[14] SHI R J, HU Z M, ZHOU Y F, et al. Research on railway freight market share based on the whole process of transport [J]. Procedia-Social and Behavioral Sciences, 2014, 138: 298-304.
[15] SHI Y, FANG X P, CHEN Z Y. Price analysis of railway freight transport under marketing mechanism [J]. Physics Procedia, 2012, 25: 2038-44.
[16] BORECKA J T, BEŠINOVIĆ N. Scheduling multimodal alternative services for managing infrastructure maintenance possessions in railway networks [J]. Transportation Research Part B: Methodological, 2021, 154: 147-74.
[17] AI Z Y, ZHANG X, WU X. Design of China-Europe intermodal container transport service network [J]. Journal of Railway Science & Engineering, 2024, 21(6).
[18] ZHANG H, LI Y, ZHANG Q P, et al. Route selection of multimodal transport based on China railway transportation [J]. Journal of Advanced Transportation, 2021, 2021(1): 9984659.
[19] SHEN X, TANG J L, ZHANG Y J, et al. Dynamic Evolution Game Strategy of Government, Power Grid, and Users in Electricity Market Demand-Side Management [J]. Mathematics, 2024, 12(20): 3249.
[20] FENG F L, ZHANG Z, CAI M X. Evolutionary Game of Multi-subject Strategies for China Railway Express Considering Subsidy Withdrawal [J]. Journal of the China Railway Society, 2023, 45(12): 47-57.
[21] YANG Y, ZHANG Z Y. Government Intervention and the Tripartite Evolutionary Game of Freight "Road to Rail" Transition [J]. Management Engineer, 2023, 28(05): 48-55.
[22] LI C B, SONG Y W, WANG J L, et al. Research on the synergistic development model of China-Europe Railway Express under the government reward-penalty mechanism [J]. Journal of Southwest Jiaotong University: 1-10.
[23] ZHANG M M, XU M Z. Co-opetition Relationship Analysis of China Railway ExpressConsidering Government Subsidy Strategy [J]. Journal of Transportation Systems Engineering and Information Technology, 2021, 21(2): 16.
[24] CHEN X, ZHU X N, ZHOU Q J, et al. Game - Theoretic Comparison Approach for Intercontinental Container Transportation: A Case between China and Europe with the B&R Initiative [J]. Journal of Advanced Transportation, 2017, 2017(1): 3128372.
[25] TAMANNAEI M, ZAREI H, RASTI-BARZOKI M. A game theoretic approach to sustainable freight transportation: Competition between road and intermodal road–rail

systems with government intervention [J]. Transportation Research Part B: Methodological, 2021, 153: 272-95.
