# Supplementary material for: Promoting the modal shift of freight from road to rail in China: An evolutionary game and simulation study
Source: PLoS One. 2025 May 23;20(5):e0320880. doi: 10.1371/journal.pone.0320880 (PMC12101862; doi:10.1371/journal.pone.0320880)
Supplement: Attachment — Submitted filename: Response to Reviewers.docx [file pone.0320880.s004.docx]

**PLoS One**

**Promoting the modal shift of freight from road to rail in China: An evolutionary game and simulation study**

*Dear Editor and Reviewers,*

We thank the editor and the reviewers for their valuable time and effort in our paper, which has helped us to improve the quality of our paper. In the following, we address each of the reviewers' comments and suggestions. We have revised our manuscript accordingly and believe that the revised version addresses all the concerns raised by the reviewers. The changes have been highlighted in yellow. We hope that our revised manuscript meets the standards of your prestigious journal.

***Reviewer #1:***

The authors appreciate your statement on our work. We have revised the paper according to your comments.

*1. I recommend that authors number their equations. This makes referencing equations easier both in the text and for reviews.*

**Response:**

We appreciate your careful review of our manuscript. We have numbered all the equations appearing in the article.

**Action:**

Please see page 11and page15-22.

*2. In subchapter 3.1, in the third and fourth equations, which give F(x) and dF(x)/dx, it is recommended to repeat the expected payoff expressions Eτ1 and Eτ2. Only the final forms for F(x) and dF(x)/dt are recommended. The observations are also valid for the corresponding equations in chapters 3.2 and 3.3*

**Response:**

We appreciate your careful review of our manuscript. We have provided the final forms of $F\left( x \right)$ and $\frac{dF\left( x \right)}{dx}$, removed redundant parts, and added numbered references to the equations for $E_{\tau_{1}}$ and $E_{\tau_{2}}$. The subsequent sections have been revised accordingly.

**Action:**

Please see page 15-20.

*3. In subchapter 3.1, please check the sign before the parenthesis in the third equation, which gives F(x) in the first square bracket.*

**Response:**

We sincerely appreciate your help in correcting this error in our manuscript. We have corrected the "+" in the equation for $F\left( x \right)$ to "-".

**Action:**

Please see page 16.

*4. At the beginning of chapter 3, we recommend specifying one or more well-known references in games and simulations of the type described below, which show the working algorithm, so that the reader can refer to them for terms of reference and developments.*

**Response:**

We sincerely appreciate the suggestion you have provided.

In this section, we introduce J. Maynard Smith’s seminal paper, Evolution and the Theory of Games. The core content of the paper focuses on how optimal strategies in situations of conflict of interest depend on the actions of others. It introduces the concept of an Evolutionarily Stable Strategy (ESS) and explores its applications in explaining animal behavior, the evolution of sex ratios, and ecosystem dynamics. At the same time, we introduce the paper *Evolutionarily Stable Strategies and Game Dynamics* by Peter D. Taylor and Leo B. Jonker. The core content of the paper explores the dynamics of evolutionary games by incorporating the concept of Evolutionarily Stable Strategy (ESS) introduced by Maynard Smith and Price. It examines the stability and asymptotic behavior of strategies in both continuous and discrete models, using non-linear differential and difference equations. The authors establish that a regular ESS is always a stable equilibrium point in continuous systems, while highlighting the differences in stability outcomes for discrete systems. Meanwhile, In the "Introduction" section, we introduce three references to illustrate the importance of numerical analysis for this study and to explain the mechanisms of similar evolutionary games and algorithms.

**Action:**

Please see page 6 and 14.

*5. In Figure 2, please explain V_A1_, V_A2_. Similar for V_B1_, V_B2_, V_C1_, V_C2_, from Figures 3 and 4.*

**Response:**

We appreciate your careful review of our manuscript. We have added relevant explanations for V_A1_, V_A2_ V_B1_, V_B2_, V_C1_, and V_C2_ following Figures 2, 3, and 4.

**Action:**

Please see page 16 , 19 and 21.

*6. It is good for payments and costs to have specified units of measurement: for example: Rf= 2000? , yuan, dollars, euros? Pag. 19). Also Ges =2000 ?, Gf= 20000 ? If they are dimensionless, specify!*

**Response:**

We sincerely appreciate your help in correcting this error in our manuscript. All monetary figures mentioned in this study are denominated in Chinese Yuan (RMB). We have added this content in the "Parameter Assignment" section of the "Simulation Analysis" chapter.

**Action:**

Please see page 28.

*7. For the Figure 6: Please explain what the variable on the abscissa, t, is. Is it the current time or a scaled time? What is the unit of measurement of the variable represented on the graphs in Figure 6? The time variable is mentioned in many places in the article. It would be useful, for a better understanding of the simulation, of the process, to explain which of the parameters that appear in the process depend on time, and, if possible, in what way (continuous, discontinuous, with jumps?).Explaining the role of time in this game can also bring estimates of the period of reaching a state of stability, of the expectations of governments to be able to give up sponsorships.*

**Response:**

We appreciate your careful review of our manuscript. We have added relevant content to the "Initial Evolutionary Path" section in the "Simulation Analysis" chapter. In the evolutionary game model, the time variable ttt represents a relative timescale, reflecting the dynamic evolution of strategies rather than actual physical time. Its unit is typically expressed as evolutionary steps or iterations, indicating strategy adjustments at different stages of system development. Moreover, simulations conducted on different timescales reveal varying evolutionary dynamics: in the short term, the system may exhibit fluctuations, while in the long term, it generally converges to a stable equilibrium point. What changes over time are not the given parameters but the probability of each decision being selected under the relevant parameters. Eventually, these probabilities will converge to a single point, which is the ESS (Evolutionarily Stable Strategy). The time scale from 0 to 1 represents the entire process of the system's evolution, starting from the current state and ultimately reaching the stable state of the ESS (Evolutionarily Stable Strategy). Time here is merely a measure to depict the evolutionary trend and does not indicate an estimated duration to reach a stable state in the actual process. By traversing the 0 to 1 time scale, this study identifies the equilibrium strategy set under the model. However, the question of when local governments in the model can cease subsidies requires further discussion and analysis under practical operational scenarios.

**Action:**

Please see page 29.

*8. The conclusions cannot be fully proven, more precisely the statements regarding the speed of stabilization, the rapidity of strategy changes and the response of the participants to the game. Therefore, we suggest an example that includes strategy changes over time, continuously or in steps. Is there an optimal strategy over time, for example for the fastest stabilization?*

**Response:**

We appreciate your careful review of our manuscript.

The description of stability speed and strategy change speed in the conclusion is derived from Figure 6. From Figure 6, we can observe that the green line representing the shippers, regardless of the initial values selected, consistently reaches "1" faster than the red line representing the local government and the blue line representing railway transport enterprises. This indicates that shippers exhibit the fastest response speed in this evolutionary game model.

In the "Stability Analysis of Equilibrium Points in the Tripartite Evolutionary Game System" section of the "Model Solution and Analysis" chapter, this study proposes four possible stability strategies, accompanied by four specific scenarios. In the "Simulation Analysis" chapter, we introduced data from references and specific station segments of China's railway system to verify that the third scenario aligns with the ESS under this model. We will gradually improve this issue in further research.

*9. The sources of numerical data in chapter 4, subchapter 4.1, must be listed in the reference list and cited normally. This is the reason for my answer to question 3. The deficiency is easy to eliminate*

**Response:**

We appreciate your careful review of our manuscript. We have included the sources of all cited data in the reference list under the "Parameter Assignment" section of the "Simulation Analysis" chapter. Specifically, for *ε* our source is the *Compilation of National Railway Statistical Data for 2023*. Due to certain specific reasons, we are unable to include this source in the reference list. We will present the reference material for this data source in the appendix of this document.

**Action:**

Please see page 28.

*10. The language can be improved (English and the academic language)*

**Response:**

We sincerely appreciate your feedback regarding language issues. Following the recommendations of the editorial team, we have utilized a professional language editing service.

**Action:**

Please refer to the appendix of this document.

**If you have any other questions or concerns, please feel free to contact the authors and we will respond and improve them seriously.**

***Reviewer #2:***

The authors appreciate your statement on our work. We have revised the paper according to your comments.

*1. Introduction: It is crucial to clearly state the following aspects in the introduction:*

*- The main research questions;*

*- The primary objectives of the study;*

*- The gaps in the literature that this study aims to address;*

*- The motivation behind the study;*

*- The innovation and novelty of the study compared to existing work.*

**Response:**

We appreciate your careful review of our manuscript. We have added the relevant content in the "Introduction" section.

**Action:**

Please see page 7 and 8.

*2. Sensitivity Analysis of Parameters: In the section on sensitivity analysis of parameters, it is recommended to include analysis of the following more critical parameters:*

*- The probability of government supervision;*

*- The penalty for shippers who apply for subsidies but fail to carry out the related freight transport as required;*

*- The government's investment subsidies for railway companies.*

**Response:**

We are very pleased that you raised questions regarding the sensitivity analysis. We have added the relevant content in the "Introduction" section. Based on your suggestions, we have added two sensitivity analyses: "sensitivity simulation analysis of the probability $\delta$ of the local government supervising shippers receiving subsidies" and "sensitivity simulation analysis of the penalty $G_{f}$ imposed on shippers for noncompliant behavior in receiving subsidies if they fail to carry out the related freight transport as required.". Regarding your mention of "The government's investment subsidies for railway companies," we did not include it after further consideration, as the funding mechanisms for railway construction are highly complex, involving multiple stakeholders, diverse financing channels, and varying regional policies.

**Action:**

Please see page 34-37.

*3. Article Structure: It is suggested to add some managerial implications after the conclusion to assist in the shift of cargo transportation from road to rail.*

**Response:**

We appreciate your careful review of our manuscript. We have added the relevant content in the "Practical Recommendations" section of the "Inspiration and Discussion" chapter.

**Action:**

Please see page 40.

*4. Grammar and Formatting: There are minor grammatical errors in the paper, and I recommend linguistic polishing to improve the readability of the manuscript.*

**Response:**

We sincerely appreciate your feedback regarding language issues. Following the recommendations of the editorial team, we have utilized a professional language editing service.

**Action:**

Please refer to the appendix of this document.

*5. Citation Formatting: There are citation formatting errors in Section 3.4 and Section 4.1. Please carefully proofread and correct these formatting issues.*

**Response:**

We sincerely appreciate your help in correcting this error in our manuscript. We have made corrections in the relevant sections of "Stability Analysis of Equilibrium Points in the Tripartite Evolutionary Game System" under the "Model Solution and Analysis" chapter and "Parameter Assignment" under the "Simulation Analysis" chapter.

**Action:**

Please see page 21 and 29.

**If you have any other questions or concerns, please feel free to contact the authors and we will respond and improve them seriously.**

***Reviewer #3:***

The authors appreciate your statement on our work. We have revised the paper according to your comments.

*1. My only concern is the discussion section. I recommend elaborating on your results by explaining how they align with or diverge from prior studies. Provide potential explanations for why your findings might support or contradict previous research and explore their implications. This will offer readers a more comprehensive understanding of the topic.*

**Response:**

We appreciate your careful review of our manuscript. We have incorporated your suggestions into the relevant content under "Alignment with and Divergence from Prior Studies" in the "Inspiration and Discussion" chapter, including the consistency with previous studies and the significance of the research.

**Action:**

Please see page 39 and 40.

**If you have any other questions or concerns, please feel free to contact the authors and we will respond and improve them seriously.**

**Appendix**

*1. Language Editing Certificate issued by AJE.*

*2. Compilation of National Railway Statistical Data for 2023*

*Because for some special reasons, I can only show you the source of the data to ensure that this data is true and reliable.*

*This material is unpublished and therefore cannot be included in the reference list.*
