# Supplementary material for: Promoting the modal shift of freight from road to rail in China: An evolutionary game and simulation study
Source: PLoS One. 2025 May 23;20(5):e0320880. doi: 10.1371/journal.pone.0320880 (PMC12101862; doi:10.1371/journal.pone.0320880)
Supplement: Attachment — Submitted filename: Response_to_Reviewers_auresp_2.docx [file pone.0320880.s005.docx]

**PLoS One**

**Promoting the modal shift of freight from road to rail in China: An evolutionary game and simulation study**

**(Second Edition)**

*Dear Editor and Reviewers,*

We thank the editor and the reviewers for their valuable time and effort in our paper, which has helped us to improve the quality of our paper. In the following, we address each of the reviewers' comments and suggestions. We have revised our manuscript accordingly and believe that the revised version addresses all the concerns raised by the reviewers. Changes made in the first revision have been highlighted in yellow, and changes made in the second revision have been highlighted in green to distinguish them. We hope that our revised manuscript meets the standards of your prestigious journal.

***Reviewer #1:***

Thank you for your positive feedback and valuable suggestions regarding our work. We will further explore and refine the relevant aspects in our future research.

Thank you again for your support and encouragement!

**If you have any other questions or concerns, please feel free to contact the authors and we will respond and improve them seriously.**

***Reviewer #2:***

The authors appreciate your statement on our work. We have revised the paper according to your comments. The yellow marked parts are the corresponding revisions.

*1. Introduction: It is crucial to clearly state the following aspects in the introduction:*

*- The main research questions;*

*- The primary objectives of the study;*

*- The gaps in the literature that this study aims to address;*

*- The motivation behind the study;*

*- The innovation and novelty of the study compared to existing work.*

**Response:**

We appreciate your careful review of our manuscript. We have added the relevant content in the "Introduction" section.

**Action:**

Please see page 7 and 8.

*2. Sensitivity Analysis of Parameters: In the section on sensitivity analysis of parameters, it is recommended to include analysis of the following more critical parameters:*

*- The probability of government supervision;*

*- The penalty for shippers who apply for subsidies but fail to carry out the related freight transport as required;*

*- The government's investment subsidies for railway companies.*

**Response:**

We are very pleased that you raised questions regarding the sensitivity analysis. We have added the relevant content in the "Introduction" section. Based on your suggestions, we have added two sensitivity analyses: "sensitivity simulation analysis of the probability $\delta$ of the local government supervising shippers receiving subsidies" and "sensitivity simulation analysis of the penalty $G_{f}$ imposed on shippers for noncompliant behavior in receiving subsidies if they fail to carry out the related freight transport as required.". Regarding your mention of "The government's investment subsidies for railway companies," we did not include it after further consideration, as the funding mechanisms for railway construction are highly complex, involving multiple stakeholders, diverse financing channels, and varying regional policies.

**Action:**

Please see page 34-37.

*3. Article Structure: It is suggested to add some managerial implications after the conclusion to assist in the shift of cargo transportation from road to rail.*

**Response:**

We appreciate your careful review of our manuscript. We have added the relevant content in the "Practical Recommendations" section of the "Inspiration and Discussion" chapter.

**Action:**

Please see page 41.

*4. Grammar and Formatting: There are minor grammatical errors in the paper, and I recommend linguistic polishing to improve the readability of the manuscript.*

**Response:**

We sincerely appreciate your feedback regarding language issues. Following the recommendations of the editorial team, we have utilized a professional language editing service.

**Action:**

Please refer to the appendix of this document.

*5. Citation Formatting: There are citation formatting errors in Section 3.4 and Section 4.1. Please carefully proofread and correct these formatting issues.*

**Response:**

We sincerely appreciate your help in correcting this error in our manuscript. We have made corrections in the relevant sections of "Stability Analysis of Equilibrium Points in the Tripartite Evolutionary Game System" under the "Model Solution and Analysis" chapter and "Parameter Assignment" under the "Simulation Analysis" chapter.

**Action:**

Please see page 22 and 29.

**If you have any other questions or concerns, please feel free to contact the authors and we will respond and improve them seriously.**

***Reviewer #3:***

The authors appreciate your statement on our work. We have revised the paper according to your comments. The green marked parts are the corresponding revisions.

*1. The authors have selected a relevant and significant topic, presenting it clearly and demonstrating a solid grasp of the fundamental concepts. They have effectively highlighted the paper's contribution, and the technical rigor of the applied processes and statistical methods remains satisfactory. However, I noticed that the changes requested regarding the discussion section were not fully addressed. Specifically, I had recommended elaborating on your results by explaining how they align with or diverge from prior studies. Additionally, I suggested providing potential explanations for why your findings might support or contradict previous research and exploring their implications. Unfortunately, these aspects remain insufficiently developed.* *I strongly encourage revisiting the discussion section to incorporate these elements. Expanding on how your findings connect to existing literature will not only enhance the robustness of your manuscript but also provide readers with a deeper and more nuanced understanding of the topic. Addressing this will greatly strengthen the overall contribution and impact of your work.*

**Response:**

We appreciate your careful review of our manuscript. Thank you for your valuable feedback and constructive suggestions. We greatly appreciate your thorough review and insightful recommendations, which have significantly helped us improve our manuscript.

Regarding your comments on the discussion section, we acknowledge that our initial revision did not fully elaborate on how our findings align with or diverge from prior studies, nor did it sufficiently explore potential explanations for these similarities and differences. In response, we have made substantial modifications to this section to strengthen the connection between our results and existing literature.

First, we have explicitly compared our findings with previous studies, particularly in terms of the participant selection in the evolutionary game model. Unlike prior research that included road transport enterprises or intermediary platforms, our study incorporates shippers as direct participants to better capture supply–demand dynamics in the transportation market. This distinction provides a more realistic depiction of decision-making processes and offers more targeted theoretical support for policy formulation and corporate strategy. In addition, we have expanded our discussion to highlight how the increasing policy support for railway transport in China aligns with global trends, as evidenced in studies on green transportation and sustainable development.

Second, we have explored potential reasons for the differences between our findings and previous research. Our results indicate that government subsidies play a crucial role in the early stages of road-to-rail transition, but their effectiveness gradually diminishes as market mechanisms take over. This observation aligns with findings on China Railway Express but differs in emphasizing the importance of well-designed subsidy exit strategies to ensure long-term market sustainability. Additionally, we discuss the observed asymmetry in the speed of strategy adjustments among stakeholders. Our findings suggest that shippers respond more quickly to policy incentives than railway transport enterprises, highlighting the necessity of synchronized policy interventions to ensure balanced progress across stakeholders. This dynamic perspective, which is often overlooked in previous static models, offers a more nuanced understanding of transportation structure adjustments.

Furthermore, we have expanded on the policy and strategic implications of our findings. We have provided a more detailed discussion on the role of government intervention in shaping market behavior and how targeted subsidies and regulatory measures can facilitate a smooth transition from government-driven to market-driven transport adjustments. At the same time, we have further clarified our study’s theoretical contributions, particularly in advancing evolutionary game modeling for transport policy analysis. Our framework captures the adaptive strategies of stakeholders over time, addressing an important research gap by integrating temporal dynamics into the study of transportation structure evolution.

We believe these revisions have significantly strengthened the discussion section, providing a clearer and more comprehensive interpretation of our findings. We sincerely appreciate your valuable suggestions, which have helped enhance the overall contribution and impact of our work.

Thank you again for your time and effort in reviewing our manuscript. We look forward to your further comments and suggestions.

**Action:**

Please see page 37-40.

**If you have any other questions or concerns, please feel free to contact the authors and we will respond and improve them seriously.**

**Appendix**

*1. Language Editing Certificate issued by AJE.*

*2. Compilation of National Railway Statistical Data for 2023*

*Because for some special reasons, I can only show you the source of the data to ensure that this data is true and reliable.*

*This material is unpublished and therefore cannot be included in the reference list.*
